# Comprehensive Comparison of the Effect of Inotropes on Cardiorenal Syndrome in Patients with Advanced Heart Failure: A Network Meta-Analysis of Randomized Controlled Trials

**DOI:** 10.3390/jcm10184120

**Published:** 2021-09-13

**Authors:** Wei-Cheng Chen, Meng-Hsuan Lin, Chieh-Lung Chen, Yi-Ching Lai, Chih-Yu Chen, Yu-Chao Lin, Chin-Chuan Hung

**Affiliations:** 1 Graduate Institute of Biomedical Sciences, China Medical University, 91 Hsueh-Shih Road, Taichung 404333, Taiwan; D20270@mail.cmuh.org.tw; 2 Division of Pulmonary and Critical Care Medicine, Department of Internal Medicine, China Medical University Hospital, 2 Yude Road, North Dist., Taichung 404332, Taiwan; D22059@mail.cmuh.org.tw (C.-L.C.); D14310@mail.cmuh.org.tw (C.-Y.C.); 3 Department of Education, China Medical University Hospital, 2 Yude Road, North Dist., Taichung 404332, Taiwan; 4 Department of Pharmacy, College of Pharmacy, China Medical University, 100 Jingmao Road, Bei-tun Dist., Taichung 406040, Taiwan; u107308201@cmu.edu.tw; 5 Department of Cardiovascular Medicine, China Medical University Hospital, 2 Yude Road, North Dist., Taichung 404332, Taiwan; D31967@mail.cmuh.org.tw; 6 School of Medicine, China Medical University, 91 Hsueh-Shih Road, Taichung 404333, Taiwan; 7 Department of Pharmacy, China Medical University Hospital, 2 Yude Road, Taichung 404332, Taiwan; 8 Department of Healthcare Administration, Asia University, 500 Lioufeng Road, Wufeng, Taichung 41354, Taiwan

**Keywords:** levosimendan, heart failure, cardiorenal syndrome, mortality, network meta-analysis

## Abstract

Prevention of cardiorenal syndrome through treatment with inotropic agents remains challenging. This network meta-analysis evaluated the safety and renoprotective effects of inotropes on patients with advanced heart failure (HF) using a frequentist random-effects model. A systematic database search was performed until 31 January 2021, and a total of 37 trials were included. Inconsistency, publication bias, and subgroup analyses were conducted. The levosimendan group exhibited significantly decreased mortality compared with the control (odds ratio (OR): 0.62; 95% confidence interval (CI): 0.46–0.84), milrinone (OR: 0.50; 95% CI: 0.30–0.84), and dobutamine (OR: 0.75; 95% CI: 0.57–0.97) groups. In terms of renal protection, levosimendan (standardized mean difference (SMD): 1.67; 95% CI: 1.17–2.18) and dobutamine (SMD: 1.49; 95% CI: 0.87–2.12) more favorably improved the glomerular filtration rate (GFR) than the control treatment did, but they did not significantly reduce the incidence of acute kidney injury. Furthermore, levosimendan had the highest P-score, indicating that it most effectively reduced mortality and improved renal function (e.g., GFR and serum creatinine level), even in patients with renal insufficiency. In conclusion, levosimendan is a safe alternative for protecting renal function on cardiorenal syndrome in patients with advanced HF.

## 1. Introduction

Heart failure (HF) affects more than 26 million people globally [1]. Renal dysfunction that occurs in 40% of patients with HF [2] is a negative predictor of the prognosis of patients with HF and plays a more crucial role than the etiology and systolic function of the heart in HF [3,4,5]. Furthermore, a decline in renal function can exacerbate the course of HF to advanced HF [6]. In particular, the complex interaction between the heart and kidneys observed in various acute and chronic disorders has recently been defined as cardiorenal syndrome [7,8]. Therapies that can ameliorate heart congestion and enhance kidney perfusion may have an effect of protecting renal function on cardiorenal syndrome in patients with advanced HF.

The use of inotropic agents is indicated for low cardiac output patients, such as those with acute decompensated HF, to increase the contractility of the heart and maintain the functions of vital organs, such as the kidneys [9,10]. Catecholamines (e.g., dopamine and dobutamine), phosphodiesterase III inhibitors (e.g., milrinone), and calcium sensitizers (e.g., levosimendan) are the most commonly used inotropic agents.

“Classic” positive inotropic drugs, such as catecholamines and phosphodiesterase III inhibitors, have been widely used. A crossover study reported that dobutamine is preferred over dopamine in HF due to its sustained inotropic response [11]. Several studies have evaluated the protective effect of low-dose dopamine on the kidneys. A meta-analysis reported that dopamine was not beneficial for renal protection in intensive care unit patients [12]. However, the Dopamine in Acute Decompensated Heart Failure study demonstrated that the dopamine group had a lower occurrence of worsening renal function [13]. However, both dobutamine and dopamine could not improve prognosis and were usually associated with increased mortality in patients with HF [14]. The Outcomes of a Prospective Trial of Intravenous Milrinone for Exacerbations of Chronic Heart Failure trial reported that milrinone led to a minor improvement in renal function but no improvement in the mortality rate [15].

Levosimendan exerts a positive inotropic effect through calcium-dependent binding and a vasodilation effect through opening ATP-sensitive potassium channels [16,17]. The Longitudinal Investigation of Depression Outcomes study indicated that compared with dobutamine, levosimendan significantly reduced the serum creatinine level and mortality in patients with HF [18]. To date, several clinical trials have examined the effects of inotropic agents on mortality and renal protection in patients with acute and chronic HF [19,20,21,22]. However, these promising agents have exhibited mixed outcomes in some clinical trials [21,23].

The effect of inotropic agents on mortality and renal protection in patients with HF remain controversial. Due to the lack of head-to-head comparison studies, data regarding various inotropic drugs are still insufficient. The network meta-analysis (NMA) provides estimates for comparisons between paired interventions that have never been evaluated in individual randomized trials. Therefore, this NMA examined the safety and renoprotective effect of inotropic drugs on patients with advanced HF.

## 2. Materials and Methods

### 2.1. Data Sources and Searches

The Preferred Reporting Items for Systematic Reviews and Meta-Analyses Protocol was conducted for this network meta-analysis (Supplementary Table S1), and the protocol of this study was approved in the PROSPERO (ID: CRD42021254941).

To evaluate the effect of inotropic agents on the treatment of patients with heart failure, we performed a comprehensive literature search. The E-databases where we searched for relevant trials were Cochrane Central Register of Controlled Trials (CENTRAL), Web of science, EMBASE, and PubMed up to January 2021. The literature search terms used for the E-databases were as follows: Heart failure AND (kidney OR renal OR cardiorenal syndrome) AND (inotropes OR calcium sensitizer OR catecholamines OR phosphodiesterase III inhibitors) AND trial.

### 2.2. Study Selection

We included studies conducted as randomized clinical trials (RCTs). The criteria for patient selection were those with advanced heart failure who were 18 years of age or older. In addition, we compared inotropic drugs with the placebo or other inotropic drugs. Eligible patients were required to be stabilized. The advanced heart failure was performed according to the following criteria [24]: (a) Severe and persistent symptoms of HF (NYHA functional class III or IV); (b) severe cardiac dysfunction; and (c) episodes of pulmonary or systemic congestion requiring high-dose diuretics or episodes of low output requiring inotropes. The following endpoints were evaluated: All-cause mortality, the value of renal-related problems, such as the glomerular filtration rate (GFR), serum creatinine (Scr), and the incidence of acute kidney injury (AKI). In addition, we excluded hemodynamically unstable patients (e.g., cardiogenic shock).

After screening titles and abstracts, two independent investigators then judged whether the trial met the eligibility criteria. Disagreements were resolved through consensus by a third reviewer. Furthermore, if any doubts about the inclusion criteria were needed to be confirmed, we contacted the corresponding authors for clarification.

### 2.3. Data Extraction and Risk of Bias Assessment

Two investigators set up a comprehensive collection form to extract data and verify the entries. The primary outcome was mortality and secondary outcomes were renal-related endpoints. If the data were available, we retained baseline characteristics of the patients and treatment options for each article.

The Revised Cochrane risk-of-bias tool for randomized trials (RoB 2.0, 22 August 2019 version, Cochrane, London, UK) were performed to assess the risk of bias as either low, some concern or high. The following individual domains were: (1) Randomization process; (2) deviations from the intended interventions; (3) missing outcome data; (4) measurement of the outcome; (5) selection of the reported result; and (6) overall risk of bias.

### 2.4. Data Synthesis and Analysis

In order to draw general conclusions from different interventions, we gathered similar treatments in a limited number of categories. Following the recommendations of cardiologists, we defined the comparator as a non-active treatment including an additional diuretic regimen or placebo. Therefore, data synthesis was carried out by classifying cardiotonic agents, and non-cardiotonic arms were regarded as the control.

We conducted a network meta-analysis (NMA) using direct and indirect estimates based on the frequentist random-effects model. For binary outcomes, we implemented this model to evaluate odds ratios (ORs) with corresponding 95% confidence intervals (CIs), while for successive results, standardized mean differences (SMDs) with corresponding 95% CIs were used. According to the DerSimonian-Laird inverse variance method, the degree of heterogeneity can be included in the research weight.

In addition, through simultaneous comparisons in the network meta-analysis, the relative ranking of a given result can be estimated as the P-score. The P-score measures the certainty that a treatment is superior to other treatments. It can range from 0% (i.e., the lower ranking of this treatment corresponds to poorer results) to 100% (i.e., the higher ranking of this treatment corresponds to better results).

We used the net split method to check the local consistency between designs (*p*-values < 0.05 mean that the method has a significant effect). The publication bias was assessed using the comparison-adjusted funnel plot (*p*-values of Egger’s Test < 0.05 mean that the funnel asymmetry is not present).

Furthermore, to explore a specific condition of heart failure patients with renal impairment, the subgroup analyses were performed in agreement with renal dysfunction in the baseline by two clinical test indicators in primary and secondary outcomes. According to the KDIGO guidelines [25], we set stage 3 chronic kidney disease (CKD) with an estimated GFR value of less than 60 mL/min/1.73 m^2^ as a cutoff value to represent renal insufficiency. In view of the limited clinical data, we chose serum creatinine (Scr) as another clinical index as a reference basis. A previous study indicated that optimal cutoff values for serum creatinine in the diagnosis of stage 3 CKD in older adults were ≥1.3 mg/dL for men and ≥1.0 mg/dL for women [26]. Among the potential risks of overestimation, we set Scr ≥ 1.5 mg/dL as the cutoff value for renal dysfunction.

If the data included in the study are not suitable for merging, we will summarize them qualitatively. The meta package in R (version 3.5.1; R project for statistical calculations, https://CRAN.R-project.org/package=netmeta, access date: 30 June 2021) was used for all of the analyses.

## 3. Results

### 3.1. Study Characteristics

**Figure 1:**
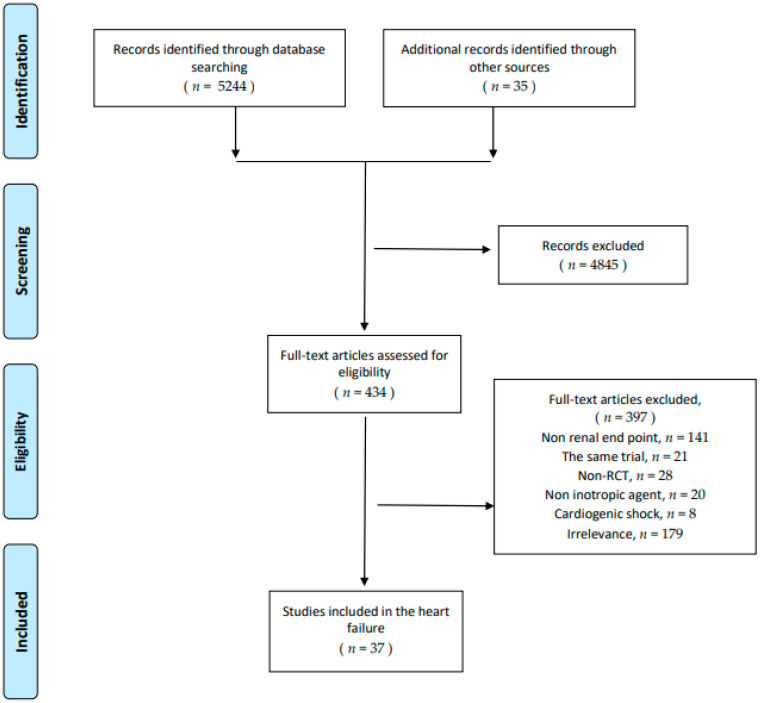
Preferred reporting items for systematic reviews and meta-analyses (PRISMA) flow diagram of randomized controlled trials, included and excluded.

Thirty seven unique RCTs and 4957 participants were included in this study (Figure 1). Most of the patients included were advanced HF. Four inotropic agents including levosimendan, milrinone, dobutamine, and dopamine were analyzed in this study. Administration doses ranged from 0.1–0.4 μg/kg/min with or without bolus 6–24 μg/kg for 10 min for levosimendan and 2.5–15 μg/kg/min with or without bolus for dobutamine. The doses for dopamine ranged from 2–5 μg/kg/min without bolus and milrinone 0.25–1 μg/kg/min with bolus. The detailed characteristics of these trials are listed in Supplementary Table S2. Furthermore, four network graphs were established for each endpoint in the analysis (Supplementary Figure S1).

The risk of bias of each included trial was assessed based on the summative assessment of the field (Supplementary Figure S2). Most of the RCTs exhibited a low risk of bias for the randomization process (37 [100%]) [13,15,18,19,23,27,28,29,30,31,32,33,34,35,36,37,38,39,40,41,42,43,44,45,46,47,48,49,50,51,52,53,54,55,56,57,58], deviations from the intended interventions (23 [62%]) [13,15,18,23,27,29,31,33,34,36,38,40,41,42,43,44,45,46,47,48,51,53,54], missing outcome data (34 [92%]) [13,15,18,19,23,27,28,29,30,31,32,33,34,35,36,37,39,40,41,42,43,44,45,46,47,48,49,52,53,54,55,56,57,58], measurement of the outcome (37 [100%]) [13,15,18,19,23,27,28,29,30,31,32,33,34,35,36,37,38,39,40,41,42,43,44,45,46,47,48,49,50,51,52,53,54,55,56,57,58], and selection of the reported results (27 [73%]) [13,15,18,19,23,27,28,29,30,31,32,34,35,36,39,40,41,42,43,44,45,46,47,48,49,53,54]. Of the 37 RCTs, 14 (38%) had some concern of bias due to the open-label problem in deviations from the intended interventions [19,28,30,32,35,37,39,49,50,52,55,56,57,58], three (38%) had some concern of bias due to the missing outcome [38,50,51], and 10 (27%) had some concern of bias due to whether the test was carried out in accordance with the plan for the selection of the reported results [33,37,38,50,51,52,55,56,57,58].

### 3.2. Overall Analysis

#### 3.2.1. Mortality

**Figure 2:**
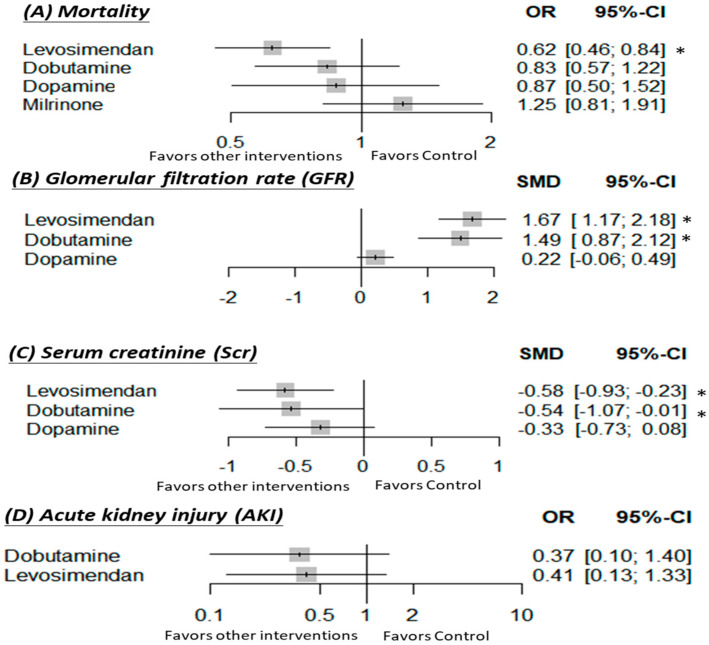
Forest plots of the overall network meta-analysis. Interventions were compared with the control for (**A**) mortality, (**B**) GFR, (**C**) Scr, and (**D**) AKI. CI: Confidence interval; OR: Odds ratio; SMD: Standardized mean difference. The control group was defined as a non-active treatment including an additional diuretic regimen or placebo. * Significant results.

For the primary outcome of mortality, 14 studies (4458 patients) provided data that could be analyzed. The overall network meta-analysis indicated that the use of levosimendan was significantly reduced mortality as compared to the use of the control (OR: 0.62; 95% CI: 0.46–0.84), milrinone (OR: 0.50; 95% CI: 0.30–0.84) or dobutamine (OR: 0.75; 95% CI: 0.57–0.97) (Table 1 and Figure 2).

#### 3.2.2. Glomerular Filtration Rate (GFR)

Eight studies (413 patients) were included in the secondary outcome of GFR. In addition, they were calculated with the modification of diet in the renal disease equation (MDRD) formula or filtration fraction (FF) x renal plasma flow (RPF). A pooled analysis of all the studies found that the use of levosimendan was associated with a significant increment of GFR as compared to the use of dopamine (SMD: 1.46; 95% CI: 0.88–2.03) or control (SMD: 1.67; 95% CI: 1.17–2.18). Moreover, the use of dobutamine significantly elevated the value of GFR as compared to the use of dopamine (SMD: 1.28; 95% CI: 0.59–1.96) or control (SMD: 1.49; 95% CI: 0.87–2.12), as well (Table 1 and Figure 2).

#### 3.2.3. Serum Creatinine (Scr)

The serum creatinine values were reported in five trials (599 patients). The results indicated that the use of levosimendan (SMD: −0.58; 95% CI: −(0.9–0.23)) or dobutamine (SMD: −0.54; 95% CI: −(1.07–0.01)) significantly decreased serum creatinine as compared to the placebo (Supplementary Table S3 and Figure 2).

#### 3.2.4. The Incidence of Acute Kidney Injury (AKI)

Data concerning the incidence of AKI were conducted in four trials (1484 patients). There was no significant difference among the use of levosimendan, dobutamine, and placebo in the outcome of AKI (Supplementary Table S3 and Figure 2).

### 3.3. Subgroup Analysis

#### 3.3.1. Mortality

The subgroup network meta-analysis of patients with renal dysfunction defined as Scr ≥ 1.5 mg/dL included four studies (1251 patients). The results exhibited that the use of levosimendan was significantly superior to the use of the control (OR: 0.13; 95% CI: 0.03–0.68), milrinone (OR: 0.11; 95% CI: 0.02–0.60) or dopamine (OR: 0.15; 95% CI: 0.02–0.95) (Table 2 and Supplementary Figure S3).

The subgroup network meta-analysis of patients with renal dysfunction defined as GFR < 60 mL/min/1.73 m^2^ included four studies (476 patients). None of the inotropic agents exhibited a significant effect on mortality (Supplementary Table S4 and Figure S4).

#### 3.3.2. Glomerular Filtration Rate (GFR)

Four studies with renal dysfunction defined as Scr ≥ 1.5 mg/dL (207 patients) performed the results of GFR based on the original renal function greater than 1.5 mg/dL. The subgroup network meta-analysis indicated that only the use of levosimendan exhibited a significant effect on the improvement of GFR in patients with renal insufficiency (Table 2 and Supplementary Figure S3).

The subgroup defined as GFR < 60 mL/min/1.73 m^2^ included seven studies (397 patients). In this subgroup network meta-analysis, compared with the control group, the use of levosimendan exhibited a significant effect on the improvement of GFR (SMD: 0.72; 95% CI: 0.28–1.16) (Supplementary Table S4 and Figure S4).

### 3.4. Finding of Ranking

**Figure 3:**
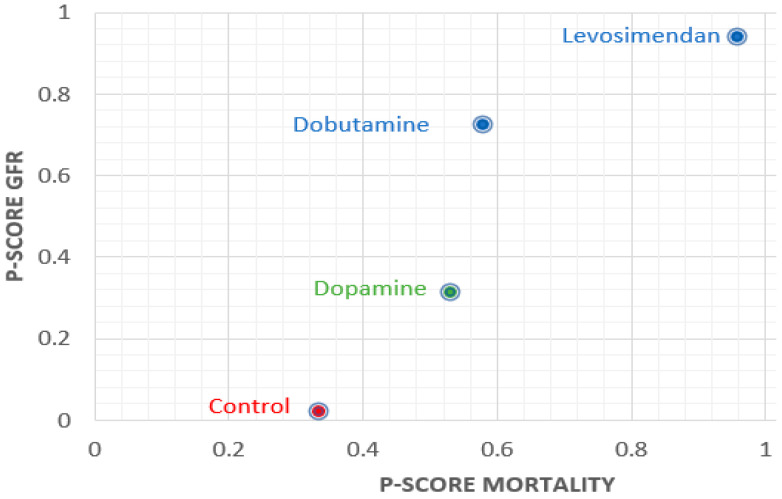
P-scores ranking plot. The X axis represents the P-score of mortality and the Y axis represents the P-score of the glomerular filtration rate (GFR). The higher P-score corresponds to better results. Therefore, the dot in the upper right corner of the chart indicates a better treatment effect.

The ranking analysis of overall analysis exhibited that the P-score of levosimendan ranked first in mortality (P-score: 0.96), GFR (P-score: 0.94), and Scr (P-score: 0.80). In addition, the subgroup with renal dysfunction defined as Scr ≥ 1.5 mg/dL of levosimendan ranked the best in mortality (P-score: 0.94) and GFR (P-score: 0.91), as well (Supplementary Table S5). The sum of ranking finding in the overall analysis for safety (P-score mortality) and renal protective efficacy (P-score GFR) is presented together in a bivariate ranking plot (Figure 3). To sum up, the results demonstrate that levosimendan had a better effect than the others.

### 3.5. Inconsistency and Publication Bias Analyses

Neither inconsistency (Supplementary Table S6) nor publication bias (Supplementary Figure S5) exhibited a significant difference. Therefore, the results were not influenced by a significant publication bias and local inconsistency.

## 4. Discussion

To the best of our knowledge, this was the first NMA that compared the effect of different inotropic agents on the mortality and three renal outcomes of patients with HF. The main finding of this study demonstrates that levosimendan was statistically significant in reducing mortality and improving renal function (e.g., GFR and Scr), even in patients with renal insufficiency. Moreover, compared with the control group, the levosimendan and dobutamine groups exhibited more significant improvement in both GFR and SCR. However, the statistic measures were not a substitute for the relative treatment effects. Indeed, none of the inotropic agents significantly reduced the incidence of AKI.

The findings of this study suggested that levosimendan was a useful and safe inotropic agent. Previous meta-analyses [14,59] have found that no inotropic agent could improve survival, except for levosimendan, in patients with cardiac disease. We attempted to elucidate mechanisms underlying the different effects of various inotropic agents on mortality. Dobutamine may cause irreversible damage to myocardial cells, whereas levosimendan may exert a protective effect on the myocardium [60,61]. Furthermore, unlike catecholamines, the hemodynamic effect of levosimendan was not attenuated by the simultaneous use of β-blockers. Therefore, the 2016 European Society of Cardiology guidelines recommended levosimendan as the first choice of drug for acute decompensated heart failure and β-blockers, if β-blockers are believed to cause hypotension and subsequent hypoperfusion (class IIb, evidence level C) [62,63].

Although we cannot interpret our results clinically, this study provided a trend towards the use of inotropic drugs for renal protection. Preclinical studies have reported that levosimendan exerts a renoprotective effect and thus can be used on the cardiorenal syndrome in patients with advanced heart failure [64,65,66]. Recently, Fedele et al. [43] and Lannemyr et al. [48] investigated the effect of dobutamine and levosimendan on GFR and reported that only levosimendan increased the GFR. In addition, Bragadottir et al. [22] indicated that although the GFR increased during the levosimendan treatment, the relationship between the renal oxygen supply and demand was not impaired. This finding is consistent with our results that although levosimendan exerted a more favorable effect on renal protection, dobutamine improved renal protection. Various renal protective mechanisms of levosimendan have been proposed: (1) Levosimendan can induce peripheral arterial and venous dilation by activating ATP-dependent potassium channels [67]; (2) target afferent arterioles cause vasodilation and exert a beneficial effect on the ultrafiltration coefficient of glomerular capillaries [21]; (3) levosimendan can increase the surface area of glomerular capillaries by inhibiting the contraction of mesangial cells mediated by angiotensin Ⅱ [64,68].

To investigate the role of levosimendan in renal impairment with limited information, we performed a subgroup analysis using two clinical indicators. Regardless of any type of clinical cutoff value, the use of levosimendan exhibited a significant improvement in GFR. Moreover, we found that compared with the control group, only the levosimendan group exhibited a significant improvement in survival in the subgroup defined as Scr ≥ 1.5 mg/dL. Although some prospective studies have reported the beneficial effect of levosimendan, it was contraindicated in patients with critical illness and renal impairment. However, a case study reported that levosimendan resulted in the rapid revival of patients with multiorgan failure [69], this result was in accordance with our findings. The prolonged effect of levosimendan might be attributable to these findings. OR-1855 and OR-1896, the active metabolites of levosimendan, had a 1.5-fold half-life (96.5 ± 19.5 h) in patients with severe renal failure with a creatinine clearance of < 30 mL/min [51]. In addition, due to the improvement in renal function after levosimendan infusion, the excretion of metabolites may be accelerated.

This study had several strengths. First, we used the exclusive methodological evaluation criteria and comprehensively extracted clinical and renal physiological results. Second, the quality of RCTs included in our study ranged from moderate to high, indicating the robustness of our results. Finally, to examine the clinical contradiction between mortality and renal protection, we used the P-score ranking to perform a quantitative analysis and rank the effectiveness and safety of inotropic drugs.

This study had some limitations that should be addressed. First, due to the fact that it was a relatively new procedure, NMA was particularly criticized for its indirect comparisons. Although a certain degree of difference between study populations is acceptable in a paired meta-analysis, this can adversely affect the results of an NMA. Therefore, we consolidated the network loop in our subgroup analysis by including a more homogeneous population, namely patients with renal impairment (Scr ≥ 1.5 mg/dL and GFR < 60 mL/min/1.73 m^2^). Moreover, no inconsistency across nodes was observed in the present study. Second, we excluded patients with acute HF complicated with cardiogenic shock from our study due to hemodynamic instability. Patients with cardiogenic shock have high heterogeneity, potential complexity, and various end-organ hypoperfusion states. In fact, levosimendan has less evidence to support cardiogenic shock. Moreover, most of the clinical trials in this study excluded patients with systolic blood pressure < 100 mmHg or heart rate >110 bpm during screening to prevent adverse events of levosimendan such as hypotension and arrhythmia. Therefore, the results in our study may differ from those for patients with cardiogenic shock. Third, most of the trials have examined the serum creatinine level to estimate the GFR, thus resulting in the risk of potential overestimation. Nevertheless, we analyzed other relevant kidney results as reference for the evaluation. In addition, we calculated a standardized mean difference rather than a mean difference from the viewpoint of generalizability [70]. Fourth, the control group received a nonactive treatment with an additional diuretic regimen or placebo. However, most of the included studies had a placebo group that received a baseline HF treatment such as angiotensin-converting enzyme inhibitors, angiotensin II receptor antagonists, β-blockers, diuretics, and amiodarone. To perform comparisons in our NMA, we included such studies to have the least effect on our results. The detailed information of all the regimens is provided in Supplementary Table S2.

## 5. Conclusions

Among the various inotropic agents, levosimendan could statistically reduce mortality and increase GFR in patients with HF. However, the statistics of the GFR values cannot be interpreted as having a clinical significance. Therefore, additional large-scale clinical trials should be conducted to confirm the role of levosimendan in protecting renal function on cardiorenal syndrome, in patients with advanced HF.

## Figures and Tables

**Table 1:** Results from the multiple-treatment comparison analyses for mortality (OR [95% Cl]) and GFR (SMD [95% Cl]) in the overall analysis.

Overall Analysis of Mortality OR (95% CI)				
**Levosimendan**	0.75 (0.57–0.97) *	0.71 (0.38–1.34)	0.50 (0.30–0.83) *	0.62 (0.46–0.84) *
0.18 (−0.19–0.55)	**Dobutamine**	0.95 (0.49–1.87)	0.67 (0.38–1.17)	0.83 (0.57–1.22)
1.46 (0.88–2.03)	1.28 (0.59–1.96)	**Dopamine**	0.70 (0.35–1.40)	0.87 (0.50–1.52)
-	-	-	**Milrinone**	1.25 (0.81–1.91)
1.67 (1.17–2.18) *	1.49 (0.87–2.12) *	0.22 (−0.06–0.49)	-	**Control**
Overall analysis of GFR SMD (95% CI)				

Comparisons between treatments should be read from left to right and the estimate is in the cell in common between the column-defining treatment and the row-defining treatment. Mortality is presented as the odds ratio (OR) with 95% confidence interval (CI), while GFR is presented as the standardized mean difference (SMD) with 95% confidence interval (CI). The overall analysis of mortality is shown in the upper right side of the table. The overall GFR analysis is shown in the lower left side of the cells. An OR smaller than 1 favors the row-defined treatment in mortality, and a SMD larger than 0 favors the column-defined treatment in the overall analysis of GFR. * Denotes *p*-value < 0.05.

**Table 2:** Results from the multiple-treatment comparison analyses for mortality (OR [95% Cl]) and GFR (SMD [95% Cl]) in the subgroup analysis (renal dysfunction defined as Scr ≥ 1.5 mg/dL).

Subgroup Analysis of Mortality OR (95% CI)				
**Levosimendan**	0.38 (0.04–3.77)	0.15 (0.02–0.95) *	0.11 (0.02–0.60) *	0.13 (0.03–0.68) *
0.18 (−0.19–0.55)	**Dobutamine**	0.40 (0.06–2.47)	0.29 (0.05–1.55)	0.35 (0.07–1.76)
-	-	**Dopamine**	0.73 (0.28–1.93)	0.88 (0.37–2.09)
-	-	-	**Milrinone**	1.20 (0.78–1.86)
0.72 (0.28–1.16) *	0.54 (−0.03–1.12)	-	-	**Control**
Subgroup analysis of GFR SMD (95% CI)				

Comparisons between treatments should be read from left to right and the estimate is in the cell in common between the column-defining treatment and the row-defining treatment. Mortality is presented as the odds ratio (OR) with 95% confidence interval (CI), while GFR is presented as the standardized mean difference (SMD) with 95% confidence interval (CI). The subgroup analysis of mortality is shown in the upper right side of the table. The subgroup GFR analysis is shown in the lower left side of the cells. An OR smaller than 1 favors the row-defined treatment in mortality, and a SMD larger than 0 favors the column-defined treatment in the overall analysis of GFR. * Denotes *p*-value < 0.05.

## Data Availability

The datasets used and/or analyzed during the current study are available from the corresponding author on reasonable request.

## Supplementary Materials

The following are available online at https://www.mdpi.com/article/10.3390/jcm10184120/s1. Table S1: PRISMA NMA Checklist of Items to Include When Reporting A Systematic Review Involving a Network Meta-Analysis; Table S2: The characteristics of included trials; Table S3: Results from the multiple-treatment comparison analyses for SCR (SMD [95% Cl]) and AKI (OR [95% Cl]) in the overall analysis; Table S4: Results from the multiple-treatment comparison analyses for mortality (OR [95% Cl]) and GFR (SMD [95% Cl]) in the subgroup analysis (renal dysfunction defined as GFR < 60 mL/min/1.73 m^2^); Table S5: P-score analyses for each outcome; Table S6: Consistency analyses of each outcome by the node-split model; Figure S1: Network plot for the included therapies; Figure S2: Risk of bias; Figure S3: Forest plots of subgroup in patients with renal dysfunction defined as Scr ≥ 1.5 mg/dL; Figure S4: Forest plots of subgroup in patients with renal dysfunction defined as GFR < 60 mL/min/1.73 m2; Figure S5: Funnel plot for each outcome.
