# Supplementary material for: Comprehensive Comparison of the Effect of Inotropes on Cardiorenal Syndrome in Patients with Advanced Heart Failure: A Network Meta-Analysis of Randomized Controlled Trials
Source: J Clin Med. 2021 Sep 13;10(18):4120. doi: 10.3390/jcm10184120 (PMC8471363; doi:10.3390/jcm10184120)
Supplement: Supplementary file 1 [file jcm-10-04120-s001.zip › jcm-1351765 - SI final.pdf]

# SUPPLEMENTARY MATERIAL

## Table S1. PRISMA NMA Checklist of Items to Include When Reporting A Systematic Review Involving a Network Meta-analysis

| Section/Topic | Item # | Checklist Item | Reported on Page # |
|---|---|---|---|
| **TITLE** | | | |
| Title | 1 | Identify the report as a systematic review *incorporating a network meta-analysis (or related form of meta-analysis).* | ***P.1*** |
| **ABSTRACT** | | | |
| Structured summary | 2 | Provide a structured summary including, as applicable:<br>**Background:** main objectives<br>**Methods:** data sources; study eligibility criteria, participants, and interventions; study appraisal; and *synthesis methods, such as network meta-analysis.*<br>**Results:** number of studies and participants identified; summary estimates with corresponding confidence/credible intervals; *treatment rankings may also be discussed. Authors may choose to summarize pairwise comparisons against a chosen treatment included in their analyses for brevity.*<br>**Discussion/Conclusions:** limitations; conclusions and implications of findings.<br>**Other:** primary source of funding; systematic review registration number with registry name. | ***P.1*** |
| **INTRODUCTION** | | | |
| Rationale | 3 | Describe the rationale for the review in the context of what is already known, *including mention of why a network meta-analysis has been conducted.*_ | ***P.2*** |
| Objectives | 4 | Provide an explicit statement of questions being addressed, with reference to participants, interventions, comparisons, outcomes, and study design (PICOS). | ***P.2*** |
| **METHODS** | | | |
| Protocol and registration | 5 | Indicate whether a review protocol exists and if and where it can be accessed (e.g., Web address); and, if available, provide registration information, including registration number. | ***P.2*** |
| Eligibility criteria | 6 | Specify study characteristics (e.g., PICOS, length of follow-up) and report | ***P.3*** |

| | | | |
|---|---|---|---|
| | | characteristics (e.g., years considered, language, publication status) used as criteria for eligibility, giving rationale. *Clearly describe eligible treatments included in the treatment network, and note whether any have been clustered or merged into the same node (with justification).*_ | |
| Information sources | 7 | Describe all information sources (e.g., databases with dates of coverage, contact with study authors to identify additional studies) in the search and date last searched. | ***P.2-3*** |
| Search | 8 | Present full electronic search strategy for at least one database, including any limits used, such that it could be repeated. | ***P.2-3*** |
| Study selection | 9 | State the process for selecting studies (i.e., screening, eligibility, included in systematic review, and, if applicable, included in the meta-analysis). | ***P.3*** |
| Data collection process | 10 | Describe method of data extraction from reports (e.g., piloted forms, independently, in duplicate) and any processes for obtaining and confirming data from investigators. | ***P.3*** |
| Data items | 11 | List and define all variables for which data were sought (e.g., PICOS, funding sources) and any assumptions and simplifications made. | ***P.3*** |
| **Geometry of the network** | **S1** | Describe methods used to explore the geometry of the treatment network under study and potential biases related to it. This should include how the evidence base has been graphically summarized for presentation, and what characteristics were compiled and used to describe the evidence base to readers. | ***P.3*** |
| Risk of bias within individual studies | 12 | Describe methods used for assessing risk of bias of individual studies (including specification of whether this was done at the study or outcome level), and how this information is to be used in any data synthesis. | ***P.3*** |
| Summary measures | 13 | State the principal summary measures (e.g., risk ratio, difference in means). *Also describe the use of additional summary measures assessed, such as treatment rankings and surface under the cumulative ranking curve (SUCRA) values, as well as modified approaches used to present summary findings from meta-analyses.* | ***P.3*** |
| Planned methods of analysis | 14 | Describe the methods of handling data and combining results of studies for each network meta-analysis. This should include, but not be limited to:<br>• *Handling of multi-arm trials;*<br>• *Selection of variance structure;*<br>• *Selection of prior distributions in Bayesian analyses; and*<br>• *Assessment of model fit.* | ***P.3*** |
| **Assessment of Inconsistency** | **S2** | Describe the statistical methods used to evaluate the agreement of direct and indirect evidence in the treatment network(s) studied. Describe efforts taken to address its presence when found. | ***P.3*** |

| | | | |
|---|---|---|---|
| Risk of bias across studies | 15 | Specify any assessment of risk of bias that may affect the cumulative evidence (e.g., publication bias, selective reporting within studies). | ***P.3*** |
| Additional analyses | 16 | Describe methods of additional analyses if done, indicating which were pre-specified. This may include, but not be limited to, the following:<br>• Sensitivity or subgroup analyses;<br>• Meta-regression analyses;<br>• *Alternative formulations of the treatment network; and*<br>• *Use of alternative prior distributions for Bayesian analyses (if applicable).*_ | ***P.3*** |
| **RESULTS†** | | | |
| Study selection | 17 | Give numbers of studies screened, assessed for eligibility, and included in the review, with reasons for exclusions at each stage, ideally with a flow diagram. | ***P.4***<br>***Figure 1*** |
| **Presentation of network structure** | **S3** | Provide a network graph of the included studies to enable visualization of the geometry of the treatment network. | ***Figure S1*** |
| **Summary of network geometry** | **S4** | Provide a brief overview of characteristics of the treatment network. This may include commentary on the abundance of trials and randomized patients for the different interventions and pairwise comparisons in the network, gaps of evidence in the treatment network, and potential biases reflected by the network structure. | ***P.4***<br>***Figure S1*** |
| Study characteristics | 18 | For each study, present characteristics for which data were extracted (e.g., study size, PICOS, follow-up period) and provide the citations. | ***P.4***<br>***Table S2*** |
| Risk of bias within studies | 19 | Present data on risk of bias of each study and, if available, any outcome level assessment. | ***P.4***<br>***Figure S2*** |
| Results of individual studies | 20 | For all outcomes considered (benefits or harms), present, for each study: 1) simple summary data for each intervention group, and 2) effect estimates and confidence intervals. *Modified approaches may be needed to deal with information from larger networks.* | ***P.4***<br>***Table S2*** |
| Synthesis of results | 21 | Present results of each meta-analysis done, including confidence/credible intervals. *In larger networks, authors may focus on comparisons versus a particular comparator (e.g. placebo or standard care), with full findings presented in an appendix. League tables and forest plots may be considered to summarize pairwise comparisons.* If additional summary measures were explored (such as treatment rankings), these should also be presented. | ***P.5-6***<br>***Table 1,2, S3***<br>***Figure 2,S3*** |
| **Exploration for inconsistency** | **S5** | Describe results from investigations of inconsistency. This may include such information as measures of model fit to compare consistency and inconsistency models, *P* values from statistical tests, or summary of | ***P.7***<br>***Table S5*** |

| | | | |
|---|---|---|---|
| | | inconsistency estimates from different parts of the treatment network. | |
| Risk of bias across studies | 22 | Present results of any assessment of risk of bias across studies for the evidence base being studied. | ***P.4 Figure S2*** |
| Results of additional analyses | 23 | Give results of additional analyses, if done (e.g., sensitivity or subgroup analyses, meta-regression analyses*, alternative network geometries studied, alternative choice of prior distributions for Bayesian analyses,* and so forth). | ***P.6-7 Figure 3,S5*** |
| **DISCUSSION** | | | |
| Summary of evidence | 24 | Summarize the main findings, including the strength of evidence for each main outcome; consider their relevance to key groups (e.g., healthcare providers, users, and policy-makers). | ***P.7-8*** |
| Limitations | 25 | Discuss limitations at study and outcome level (e.g., risk of bias), and at review level (e.g., incomplete retrieval of identified research, reporting bias). *Comment on the validity of the assumptions, such as transitivity and consistency. Comment on any concerns regarding network geometry (e.g., avoidance of certain comparisons).* | ***P.8-9*** |
| Conclusions | 26 | Provide a general interpretation of the results in the context of other evidence, and implications for future research. | ***P.9*** |
| **FUNDING** | | | |
| Funding | 27 | Describe sources of funding for the systematic review and other support (e.g., supply of data); role of funders for the systematic review. This should also include information regarding whether funding has been received from manufacturers of treatments in the network and/or whether some of the authors are content experts with professional conflicts of interest that could affect use of treatments in the network. | ***P.9*** |

## Table S2. The characteristics of included trials

| Study | Inclusion criteria | n | Drug | Administration Dose | Scr (GFR) baseline[a] | EF baseline[a] | Study design | Outcomes (follow up) | Results |
|---|---|---|---|---|---|---|---|---|---|
| Slawsky (2000) | Patients with decompensated HF (NYHA III-IV and LVEF ≤30%) | 98 | **Levo** | **loading dose: 6 μg/kg for 10 mins maintaince dose: 0.1-0.4μg/kg/min for 50mins At hourly intervals, a repeat bolus was given, and the infusion rate was increased by increments of 0.1 mg/kg.** | NA | 21 ± 1 | randomized, double-blind study | Mortality (NA) | Levosimendan caused rapid dose-dependent improvement in hemodynamic function in patients with decompensated heart failure. |
| | | 48 | **Place** | | NA | 20 ± 1 | | | |
| Nieminen (2000) | Patient with chronic HF (NYHA II-IV and LVEF ≤40%) | 23 | **Levo** | **loading dose: 3, 6, 12, 24 μg/kg for 10 mins maintaince dose: 0.05, 0.1 ,0.2 ,0.4, 0.6 μg/kg/min for 24 hrs** | NA | 26 ± 2 | randomized, study | Mortality (NA) | There were no deaths during the treatment day, but two patients died during follow-up. One received dobutamine and the other received levosimendan. |
| | | 20 | **Dobu** | **maintaince dose: 6 μg/kg/min for 24 hrs** | NA | 24 ± 2 | | | |
| | | 21 | **Place** | | NA | 27± 2 | | | |
| Follath (2002) (LIDO study) | Patients with severe low-output HF (EF ≤ 35%, CI < 2.5L/min/m2, and PCWP > 15 mm Hg) | 103 | **Levo** | **loading dose: 24 μg/kg for 10 mins maintaince dose: 0.1μg/kg/min for 24 hrs** | NA | NA | randomized, double-blind, multicentre study NCT00219388 | Mortality (3m) | Levosimendan showed better haemodynamic performance and lower mortality than dobutamine in patients with severe, low-output heart failure. |
| | | 100 | **Dobu** | **maintaince dose: 5 μg/kg/min for 24 hrs The infusion rate was doubled if the response was inadequate at 2 h.** | NA | NA | | | |
| Moiseyev VS (2002) (RUSSLAN) | Patients with left ventricular failure due to an acute myocardial infarction | 402 | **Levo** | **loading dose: 6-24 μg/kg for 10 mins maintaince dose: 0.1-0.4μg/kg/min for 5 hrs and 50 mins** | NA | NA | randomized, double-blind, multicentre study | Mortality (3m) | Levosimendan reduced the risk of worsening heart failure and death in patients with left ventricular failure complicating acute myocardial infarction. |
| | | 102 | **Place** | | NA | NA | | | |

**Table S2. The characteristics of included trials (continued)**

| Study | Inclusion criteria | n | Drug | Administration Dose | Scr (GFR) baseline[a] | EF baseline[a] | Study design | Outcomes (follow up) | Results |
|---|---|---|---|---|---|---|---|---|---|
| Adamopoulos (2006) | Patients with acutely decompensated chronic HF (NYHA III-IV and LVEF ≤30% and CI <2.5L/ml/m2) | 23 | **Levo** | **loading dose: 6 μg/kg for 10 mins maintaince dose: 0.1μg/kg/min for 24 hrs** | NA | 24 ± 2 | randomized, open-labeled study | Mortality (2m) | Event-free survival was significantly longer in the levosimendan group than palcebo and dobutamine. |
| | | 23 | **Dobutamin** | **maintaince dose: 5 μg/kg/min for 24 hrs The infusion rate was doubled if the response was inadequate at 2 h.** | NA | 25 ± 1 | | | |
| | | 23 | **Place** | | NA | 27 ± 1 | | | |
| Flevari P (2006) | Patients with decompensated advanced HF (EF ≤30%, NYHA class III or IV) and secondary to ischemic or dilated cardiomyopathy | 30 | **Levo** | **loading dose: NA maintaince dose: 0.1μg/kg/min for 24 hrs** | 1.5 ± 0.1 | 22 ± 1.4 | randomized, double-blind study | Scr (24hr) | Levosimendan increases nonsustained ventricular arrhythmias. At the same time, it is associated with improvements in cardiac function and neurohormonal response. |
| | | 15 | **Place** | | 1.4 ± 0.2 | 24 ± 2.3 | | | |
| Parissis JT (2006) | Patients with decompensated chronic HF (NYHA III-IV and LVEF ≤30%) | 17 | **Levo** | **loading dose: 6 μg/kg for 10 mins maintaince dose: 0.1μg/kg/min for 24 hrs** | 1.3 ± 0.3 | 22 ± 4 | randomized, open-labeled study | Scr (24hr) | Serial levosimendan treatments improved left ventricular performance and modulated neurohormonal and immune activation beneficially in patients with advanced heart failure, without increasing myocardial injury |
| | | 8 | **Place** | | 1.4 ± 0.2 | 23 ± 4 | | | |

**Table S2. The characteristics of included trials (continued)**

| Study | Inclusion criteria | n | Drug | Administration Dose | Scr (GFR) baseline[a] | EF baseline[a] | Study design | Outcomes (follow up) | Results |
|---|---|---|---|---|---|---|---|---|---|
| Trikas(2006) | Patients with decompensated HF (NYHA III-IV and LVEF ≤35%) | 17 | **Levo** | **loading dose: 6 μg/kg for 10 mins maintaince dose: 0.1-0.4μg/kg/min for 24 hrs over a 30-day period** | NA | NA | randomized, double-blind study | Mortality (1m) | Levosimendan decreases more f proinflammatory cytokines and lower mortality than placebo. |
| | | 17 | **Place** | | NA | NA | | | |
| Mebazaa A (2007) (SURVIVE) | Patients with acute decompensated HF (LVEF ≤30%, NYHA class III or IV) | 664 | **Levo** | **loading dose: 12 μg/kg for 10 mins maintaince dose: 0.1μg/kg/min for 50 mins** | NA | 24 ± 5 | randomized, double-blind, multicentre study NCT00348504 | mortality, AKI (3m) | Levosimendan did not significantly reduce all-cause mortality at 180 days or affect any secondary clinical outcomes compared with dobutamine. |
| | | 663 | **Dobu** | **maintaince dose: 5 μg/kg/min for 24 hrs** | NA | 24 ± 5 | | | |
| Yilmaz MB (2007) | Patients with acutely decompensated HF (NYHA III-IVand LVEF ≤40%) | 58 | **Levo** | **loading dose: NA maintaince dose: 0.1-0.2μg/kg/min for 24 hrs** | 1.58 ± 0.56 (51.5) | 20 ± 3 | randomized, double-blind study | GFR, Urine (24hr) | Levosimendan seems to provide beneficial effects in 24nr-GFR and urine output compared to dobutamine in patients with heart failure. |
| | | 30 | **Dobu** | **maintaince dose: 5 μg/kg/min for 24 hrs** | 1.41 ± 0.41 (54.7) | 20 ± 4 | | | |
| Zemljic G (2007) | Patients with advanced chronic HF (NYHA III-IV, awaiting cardiac transplantation) | 20 | **Levo** | **loading dose: 12 μg/kg for 10 mins maintaince dose: 0.1μg/kg/min for 24 hrs** | 1.92 ± 0.13 | 28 ± 18 | randomized, study | Scr (3m) | Levosimendan improves long-term renal function (Scr) in advanced chronic heart failure patients awaiting cardiac transplantation. |
| | | 20 | **Place** | | 1.91 ± 0.12 | 27 ± 17 | | | |

**Table S2. The characteristics of included trials (continued)**

| Study | Inclusion criteria | n | Drug | Administration Dose | Scr (GFR) baseline[a] | EF baseline[a] | Study design | Outcomes (follow up) | Results |
|---|---|---|---|---|---|---|---|---|---|
| Yilmaz (2009) | Patients with acutely decompensated HF (NYHA III-IV and LVEF ≤35%) | 27 | **Levo** | **loading dose: NA**<br>**maintaince dose: 0.1-0.2μg/kg/min for 24 hrs** | 1.35 ± 0.37 | 22 ± 6 | randomized, study | Scr, Urine (24hr) | Levosimendan improved both 24-h urine output and creatinine, whereas dobutamine only showed significant improvement in urine output. |
| | | 13 | **Dobu** | **maintaince dose: 5 μg/kg/min for 24 hrs** | 1.27 ± 0.31 | 24 ± 6 | | | |
| Kleber(2009) | Patient with right HF due to pulmonary hypertension( NYHA III-IV, mRAP ≥4 mm Hg, mPAP ≥30 mm) | 18 | **Levo** | **loading dose: 12 μg/kg for 10 mins**<br>**maintaince dose: 0.1μg/kg/min for 50 mins and thereafter increase to 0.2μg/kg/min for 23 hrs**<br>**4 times at 2-week intervals as a continuous infusion of 0.2 μg/kg/min for 6 hours.** | NA | NA | randomized, double-blind study | mortality,AKI (2m) | One patient in placebo group died 10 days after completing the 24-hour infusion as a result of progression of heart failure and acute kidney failure. |
| | | 10 | **Place** | | NA | NA | | | |
| Iyisoy A (2010) | Patients with acute decompensated HF (EF ≤35%, NYHA class III or IV) | 20 | **Levo** | **loading dose: 12 μg/kg for 10 mins**<br>**maintaince dose: 0.1μg/kg/min for 24 hrs** | 1.6 ± 0.44 | 24 ± 15 | randomized, study | Scr (24hr) | Levosimendan and dobutamine showed similar serum creatinine level after 24 hrs. |
| | | 20 | **Dobu** | **maintaince dose: 5 μg/kg/min for 24 hrs** | 1.6 ± 0.37 | 27 ± 16 | | | |
| Pasqui(2011) | Patients with advanced HF | 35 | **Levo** | **loading dose: 6 μg/kg for 10 mins**<br>**maintaince dose: 0.1μg/kg/min for 24 hrs** | NA | NA | randomized, double-blind study | Mortality (7d) | Levosimendan and placebo showed similar mortality rate. |
| | | 32 | **Place** | | NA | NA | | | |

## Table S2. The characteristics of included trials (continued)

| Study | Inclusion criteria | n | Drug | Administration Dose | Scr (GFR) baseline[a] | EF baseline[a] | Study design | Outcomes (follow up) | Results |
|---|---|---|---|---|---|---|---|---|---|
| Bonios MJ (2012) | Patients with decompensated, end stage chronic HF (NYHA class IV) and refractory to standard therapy weaned from an inotrope infusion during 72 h from their initial hospitalization | 19 | **Levo** | **loading dose: NA**<br>**maintaince dose: 0.6μg/kg/min for 6 hrs once weekly over a 6-month period** | 1.5 ± 0.5 | 23.6 ± 7.8 | randomized, open-labeled study | mortality, Scr (3m) | In patients with refractory end-stage heart failure, intermittent administration of levosimendan conferred survival and hemodynamic benefits in comparison to dobutamine, alone or in combination with levosimendan. |
| | | 12 | **Dobu** | **maintaince dose: 10 mg/kg/min for 6 hrs once weekly over a 6 months period** | 1.2 ± 0.4 | 22.3 ± 5.5 | | | |
| Llorens(2012) | Patients with decompensated acute HF (NYHA III-IV) | 25 | **Levo** | **loading dose: 6 μg/kg for 10 mins**<br>**maintaince dose: 0.1μg/kg/min for 24 hrs** | 1.3 ± 0.4 | NA | randomized, triple-blind, single-center study EudraCT 2007-002447-25 | mortaltiy (6m) | Levosimendan produces no significant differences in readmission or mortality rates compared with placebo. |
| | | 20 | **Place** | | 1.3 ± 0.4 | NA | | | |
| Hou ZQ (2013) | Patients with decompensated HF (LVEF<40%) and renal dysfunction (eGFR 15–89 mL/min/1.73 m2) | 33 | **Levo** | **loading dose: 12 μg/kg for 10 mins**<br>**maintaince dose: 0.05μg/kg/min or 0.1μg/kg/min or 0.2μg/kg/min for 24 hrs** | 1.66 ± 0.36 (39.4) | 32.7 ± 3.6 | randomized, double-blind study | GFR (3d), urine (24hr) | A 24-h infusion with levosimendan transiently improved the renal dysfunction compared with placebo. |
| | | 33 | **Place** | | 1.64 ± 0.38 (40.4) | 33.6 ± 2.9 | | | |

**Table S2. The characteristics of included trials (continued)**

| Study | Inclusion criteria | n | Drug | Administration Dose | Scr (GFR) baseline[a] | EF baseline[a] | Study design | Outcomes (follow up) | Results |
|---|---|---|---|---|---|---|---|---|---|
| Husebye (2013) (LEAF) | Patients with acute HF following primary PCI treated acute STEMI | 30 | **Levo** | **loading dose: NA maintaince dose: 0.2μg/kg/min for 1 hr thereafter decrease to 0.1μg/kg/min for 24 hrs** | NA | 43(38-49) | randomized, double-blind, single-center study NCT00324766 | mortality (6m) | Levosimendan improved contractility in post-ischaemic myocardium without any increase in arrhythmias and mortality. |
| | | 31 | **Place** | | NA | 40(33-47) | | | |
| Francesco (2014) | Patients with acute decompensated HF (wedge pressure >20mmHg and EF <40%) and moderate renal impairment (30<GFR<90) | 14 | **Levo** | **loading dose: 6 μg/kg for 10 mins maintaince dose: 0.1μg/kg/min for 24 hrs** | 1.76 ± 0.37 (38.71) | 27.07 ± 7.8 | randomized, double-blind, single-center study NCT00527059 | Scr, GFR,Urine (3d) | Levosimendan has an immediate renoprotective effect, mediated by an increase in renal blood flow, due to a selective renal arterial and venous vasodilating action. |
| | | 7 | **Place** | | 1.68 ± 0.27 (43.33) | 31.4 ± 5.56 | | | |
| Jia (2014) | Patients with HF complicated by acute myocardial infarction (EF ≤40%) | 80 | **Levo** | **loading dose: 6 μg/kg for 10 mins maintaince dose: 0.1μg/kg/min for 24 hrs** | NA | 28.5 ± 5.7 | randomized, single-blind, single-center study | Mortality (6m) | Short-term intravenous infusion of levosimendan appears to be more effective than placebo for treating patients with heart failure complicated by AMI. |
| | | 80 | **Place** | | NA | 29.9 ± 5.3 | | | |
| Jia (2015) | Patients with acute decompensated HF (EF ≤35%, NYHA class III or IV) | 30 | **Levo** | **loading dose: NA maintaince dose: 0.1μg/kg/min for 24 hrs** | 1.09 ± 0.38 | 27.8 ± 6.8 | randomized, single-blind study | Motality (3m), AKI (5d) | Levosimendan and nesiritide was superior to placebo and single-drug therapies in terms of improvements in clinical conditions during the early stages of therapy. |
| | | 30 | **Place** | | 1.25 ± 0.58 | 29.6 ± 6.1 | | | |

**Table S2. The characteristics of included trials (continued)**

| Study | Inclusion criteria | n | Drug | Administration Dose | Scr (GFR) baseline[a] | EF baseline[a] | Study design | Outcomes (follow up) | Results |
|---|---|---|---|---|---|---|---|---|---|
| Mushtaq (2015) | Patients with advanced chronic HF (EF ≤35%, NYHA class III or IV) | 23 | **Levo** | **loading dose: NA maintaince dose: 0.05-0.2 μg/kg/min for 48hrs** | 1.5 ± 0.5 | 25 ± 6 | randomized, double-blind, single-center study NCT02261948 | Scr (24hr) | Levosimendan treatment significantly improves peak VO2 and reduces VE/VCO2 slope and BNP in patients with advanced chronic heart failure |
| | | 19 | **Place** | | 1.7 ± 0.6 | 25 ± 7 | | | |
| Comín-Colet (2018) (LION-HEART) | Patients with advanced HF (EF ≤35%, NYHA class III or IV) | 48 | **Levo** | **loading dose: NA maintaince dose: 0.2μg/kg/min for 6 hrs every 2 weeks over a 12-week period** | 1.3 ± 0.4 (59) | 27 ± 9 | randomized, double-blind, multicentre study NCT01536132 | mortaltiy,AKI (3m) | Intermittent administration of levosimendan reduced plasma concentrations of NT-proBNP, worsening of HRQoL and hospitalisation for heart failure. |
| | | 21 | **Place** | | 1.4 ± 0.3 (49) | | | | |
| Lannemyr (2018) | Patients with chronic heart failure (LVEF <40%) and impaired renal function (GFR < 80 mL/min/1.73 m2) | 16 | **Levo** | **loading dose: 12 μg/kg for 10 mins maintaince dose: 0.1μg/kg/min for 65 hrs** | 1.62 ± 0.42 (49.4) | 27.2 ± 8.0 | randomized, double-blind, single-center study NCT02133105 | GFR (3d) | Levosimendan increases glomerular filtration rate to a greater extent than dobutamine in patients with chronic heart failure and impaired renal function. |
| | | 16 | **Dobu** | **maintaince dose:5 μg/kg/min for 10 mins and thereafter increase to 7.5 μg/kg/min for 65 mins** | 1.38 ± 0.35 (55.3) | 26.0 ± 8.1 | | | |
| Wang (2019) | Patients with acutely decompensated HF | 30 | **Levo** | **loading dose: 6-12 μg/kg for 10 mins maintaince dose: 0.1μg/kg/min for 24 hrs** | 1.24 ± 0.45 | 31.2 ± 6.32 | randomized, open-label, | Mortality (1m), | Levosimendan seems to provide more beneficial effects to improve |

| | | | | | | | | | |
|---|---|---|---|---|---|---|---|---|---|
| | (NYHA III-IVand LVEF ≤45%) | 29 | **Place** | | 1.19 ± 0.4 | 33.59 ± 5.76 | single-center study NCT02133105 | SCR (24hr), Urine (24hr) | RV function, along with a decrease in pulmonary pressure. |

**Table S2. The characteristics of included trials (continued)**

| Study | Inclusion criteria | n | Drug | Administration Dose | Scr (GFR) baseline[a] | EF baseline[a] | Study design | Outcomes (follow up) | Results |
|---|---|---|---|---|---|---|---|---|---|
| Nanas(2004) | Patient with decompensated chronic HF (NYHA IV) | 16 | **Dobu** | **maintaince dose: 10 µg/kg/min for 8 hrs biweekly** | 1.7 ± 0.9 | 22.7 ± 5.8 | randomized, double-blind study | Mortality (12m), Scr (3m) | Long-term intermittent dobutamine infusion combined with amiodarone improved the survival of patients with advanced HF. |
| | | 14 | **Place** | | 1.7 ± 0.9 | 23.9 ± 5 | | | |
| Robinson (1994) | Patients with congestive HF in elderly | 8 | **Dopa** | **maintaince dose: 2.5 µg/kg/min** | NA | NA | randomized, double-blind study | Scr,GFR,Urine (24hr) | Dopamine at low dosage was not shown to benefit elderly patients with resistant congestive cardiac failure. |
| | | 8 | **Place** | | NA | NA | | | |
| Varriale (1997) | Patients with congestive HF (NYHA III-IV) and renal insufficiency ( BUN ≥ 225 mg/dl and Scr ≥ 1.5 mg/dl) | 10 | **Dopa** | **maintaince dose: 2 µg/kg/min** | 1.97 ± 0.2 | 29.5 ± 8.6 | randomized study | Scr, Urine (24hr) | The renal-protective effect of low-dose dopamine in the setting of congestive heart failure and vigorous diuresis is supported by this study. |
| | | 10 | **Diure** | **Bumetanide 1mg BID** | 1.84 ± 0.2 | 27 ± 9.6 | | | |
| Giamouzis (2010) (DAD-HF) | Patients with acutely decompensated HF | 30 | **Dopa** | **maintaince dose: 5 µg/kg/min** | 1.23 ± 0.33 (56.5) | 35.5 ± 11.2 | randomized, double-blind study NCT00937092 | mortality (2m), Scr,GFR,Urine (24hr) | The combination of low-dose furosemide and low-dose dopamine is equally effective as high-dose furosemide but associated with improved renal function profile and potassium homeostasis. |
| | | 30 | **Diure** | **high dose Furosemide** | 1.22 ± 0.43 (59.9) | 35.1 ± 13.1 | | | |

## Table S2. The characteristics of included trials (continued)

| Study | Inclusion criteria | n | Drug | Administration Dose | Scr (GFR) baseline[a] | EF baseline[a] | Study design | Outcomes (follow up) | Results |
|---|---|---|---|---|---|---|---|---|---|
| Chen (2013) (ROSE) | Patients with acute HF and renal dysfunction (GFR = 15-60 mL/min/1.73 m2) | 122 | **Dopa** | **maintaince dose: 2 μg/kg/min** | 1.59 ± 0.5 (45.5) | 35 ± 7.25 | randomized, double-blind, multicentre study NCT01132846 | Mortality (3m), Urine (24hr) | Neither low-dose dopamine nor low-dose nesiritide enhanced decongestion or improved renal function in patients with acute HF and renal dysfunction. |
| | | 119 | **Diure** | | 1.64 ± 0.5 (44.5) | 30 ± 7.5 | | | |
| Triposkiadis (2014) (DAD-HF II) | Patients with acutely decompensated HF | 56 | **Dopa** | **maintaince dose: 4-5 μg/kg/min** | 1.23 ± 0.2 (48.7) | 30 ± 4.5 | randomized, single -blind, multicentre study NCT01060293 | mortality (12m), Scr,GFR,Urine (24hr) | There were no significant differences between high- vs. low-dose furosemide infusion; the addition of low-dose dopamine infusion was not associated with any beneficial effects. |
| | | 50 | **Diure** | **high dose Furosemide** | 1.22 ± 0.1 (53.1) | 32 ± 5 | | | |
| Kamiya (2015) | Patients with acute HF and Diure resistance to natriuretic peptide | 12 | **Dopa** | **maintaince dose: 1-3 μg/kg/min** | 0.99 ± 0.34 (59.4) | 37.5 ± 15.6 | randomized, open-labeled study | Scr,GFR,Urine (24hr) | It might not have a harmful effect on renal function and effects of diuresis and symptom relief in added dopamine and added furosemide groups. |
| | | 12 | **Diure** | **low dose Furosemide** | 0.89 ± 0.24 (64.3) | 42.8 ± 19.7 | | | |
| Biddle (1987) | Patients with HF (NYHA III-IV) | 40 | **Milri** | **loading dose: 50-75 μg/kg**<br>**maintaince dose: 0.5-1.0 μg/kg/min** | NA | NA | randomized study | mortality (NA) | Milrinone and dobutamine elicited similar beneficial |

| | | | | | | | | | |
|---|---|---|---|---|---|---|---|---|---|
| | secondary to either ischemic or dilated cardiomyopathy | 39 | **Dobu** | **maintaince dose: 2.5 - 15 μg/kg/min** | NA | NA | | | hemodynamic results with relatively few adverse effects. |

**Table S2. The characteristics of included trials (continued)**

| Study | Inclusion criteria | n | Drug | Administration Dose | Scr (GFR) baseline[a] | EF baseline[a] | Study design | Outcomes (follow up) | Results |
|---|---|---|---|---|---|---|---|---|---|
| Karlsberg (1996) | Patients with acute congestive HF (NYHA III-IV) following acute myocardial infarction | 16 | **Milri** | **loading dose: 50 μg/kg** **maintaince dose: 0.25-0.75μg/kg/min** | NA | NA | randomized, open-labeled study | mortality (NA) | The short-term infusion of milrinone may have a role in the management of congestive heart failure following acute myocardial infarction. |
| | | 14 | **Dobu** | **maintaince dose: 2.5 - 15 μg/kg/min** | NA | NA | | | |
| Aranda (2002) | Patients with decompensated HF awaiting cardiac transplantation | 19 | **Milri** | **maintaince dose: 0.25-0.75 μg/kg/min** | NA | NA | randomized, open-labeled study | mortality (followed until death, transplantation or cardiac support) | Both dobutamine and milrinone can be used successfully as pharmacologic therapy for a bridge to heart transplantation. |
| | | 17 | **Dobu** | **maintaince dose: 2.5 - 10μg/kg/min** | NA | NA | | | |
| Cuffe (2002) (OPTIME-CHF) | Patients with acute exacerbation of chronic HF (NYHA III-IV, mean LVEF 23%) | 477 | **Milri** | **loading dose: 50 μg/kg** **maintaince dose: 0.375-0.75 μg/kg/min for 48-72 hrs** | 1.4 ± 0.5 | NA | randomized, double-blind, multicentre study | mortality (2m) | These results do not support the routine use of intravenous milrinone as an adjunct to standard therapy in chronic heart failure. |
| | | 472 | **Place** | | 1.5 ± 0.5 | NA | | | |

The data are presented as mean± SD.

AKI: Acute kidney injury; EF: Left Ventricular Ejection Fraction; GFR: Glomerular Filtration Rate; HF: Heart failure; Scr: Serum Creatinine; m:months; d:days; hr: hours

[a] the unit of value: Scr(mg/dl), GFR (ml/min/1.73m$^2$), EF(%)

**Table S3. Results from the multiple-treatment comparison analyses for SCR (SMD [95%CI]) and AKI (OR[95%CI]) in overall analysis**

| | | | |
|---|---|---|---|
| Overall analysis of SCR SMD (95% CI) | | | |
| **Levosimendan** | -0.04 (-0.50 - 0.42) | -0.26 (-0.79 - 0.28) | -0.58 (-0.93 - -0.23)* |
| 1.09 (0.61 - 1.97) | **Dobutamine** | -0.22 (-0.88 - 0.45) | -0.54 (-1.07 - -0.01)* |
| - | - | **Dopamine** | -0.33 (-0.73 - 0.08) |
| 0.41 (0.13 - 1.33) | 0.37 (0.10 - 1.40) | - | **Control** |
| Overall analysis of AKI OR (95% CI) | | | |

Comparisons between treatments should be read from left to right and the estimate is in the cell in common between the column-defining treatment and the row-defining treatment. The overall analysis of Scr presented as SMD with 95% CI is shown in the upper right of the table. The AKI presented as OR with 95% CI is shown in the lower left of the cells. A SMD smaller than 0 favors the row-defined treatment in SCR, while a OR smaller than 1 favors the column-defined treatment in AKI. * Denotes p-value < 0.05.

**Table S4. Results from the multiple-treatment comparison analyses for mortality (OR [95%CI]) and GFR (SMD [95%CI]) in subgroup analysis (renal dysfunction defined as GFR< 60 ml/min/1.73m2)**

| | | | |
|---|---|---|---|
| Subgroup analysis of mortality OR (95% CI) | | | |
| **Levosimendan** | - | 0.85 (0.25 - 2.82) | 0.74 (0.25 - 2.16) |
| 0.18 (-0.19 - 0.55) | **Dobutamine** | - | - |
| 0.52 (-0.01 - 1.05) | 0.34 (-0.30 - 0.99) | **Dopamine** | 0.87 (0.50 - 1.52) |
| 0.72 ( 0.28 - 1.16)* | 0.54 (-0.03 - 1.12) | 0.20 (-0.08 - 0.49) | **Control** |
| Subgroup analysis of GFR SMD (95% CI) | | | |

Comparisons between treatments should be read from left to right and the estimate is in the cell in common between the column-defining treatment and the row-defining treatment. Mortality is presented as odds ratio (OR) with 95% credible interval (CI), while GFR is presented as standardized mean difference (SMD) with 95% credible interval (CI). The overall analysis of mortality is shown in the upper right of the table. The overall GFR analysis is shown in the lower left of the cells. An OR smaller than 1 favors the row-defined treatment in mortality, and a SMD larger than 0 favors the column-defined treatment in overall analysis of GFR. * Denotes p-value < 0.05.

## Table S5. P-score analyses for each outcome

| Rank | Mortality | | GFR | | Scr | AKI |
|---|---|---|---|---|---|---|
| | overall | subgroup | overall | subgroup | overall | overall |
| **1** | Levosimendan (0.96) | Levosimendan (0.94) | Levosimendan (0.94) | Levosimendan (0.91) | Levosimendan (0.80) | Dobutamine (0.77) |
| **2** | Dobutamine (0.58) | Dobutamine (0.72) | Dobutamine (0.72) | Dobutamine (0.57) | Dobutamine (0.72) | Levosimendan (0.66) |
| **3** | Dopamine (0.53) | Dopamine (0.38) | Dopamine (0.31) | Control (0.02) | Dopamine (0.46) | Control (0.07) |
| **4** | Control (0.33) | Control (0.32) | Control (0.02) | | Control (0.03) | |
| **5** | Milrinone (0.10) | Milrinone (0.14) | | | | |

Rank probability of best therapy and P-score for different inotropic therapies in the overall heart failure population for evaluated endpoints. P-score is 1 for a treatment considered to be the best, and 0 for the worst. The subgroup with renal dysfunction defined as Scr≥ 1.5 mg/dL.

**Table S6. Consistency analyses of each outcome by node-split model**

| Comparisons | Direct | Indirect | z | p-value |
|---|---|---|---|---|
| **Mortality** | | | | |
| **Dobutamine:Levosimendan** | 1.3753 | 0.8659 | 0.8003 | 0.4235 |
| **Dobutamine:Milrinone** | 0.3471 | 0.7125 | -0.7153 | 0.4744 |
| **Dobutamine:Control** | 0.8439 | 0.8316 | 0.0258 | 0.9794 |
| **Levosimendan: Control** | 0.6407 | 0.4374 | 0.6532 | 0.5136 |
| **Milrinone: Control** | 1.2035 | 2.4708 | -0.7153 | 0.4744 |
| **Serum creatinine** | | | | |
| **Levosimendan: Dobutamine** | 0.1291 | -0.6691 | 1.3846 | 0.1662 |
| **Levosimendan: Control** | -0.6691 | 0.1291 | -1.3846 | 0.1662 |
| **Dobutamine: Control** | 0.0000 | -0.7982 | 1.3846 | 0.1662 |

p-value less than 0.05 represented presence of inconsistency. * Denotes p-value < 0.05.

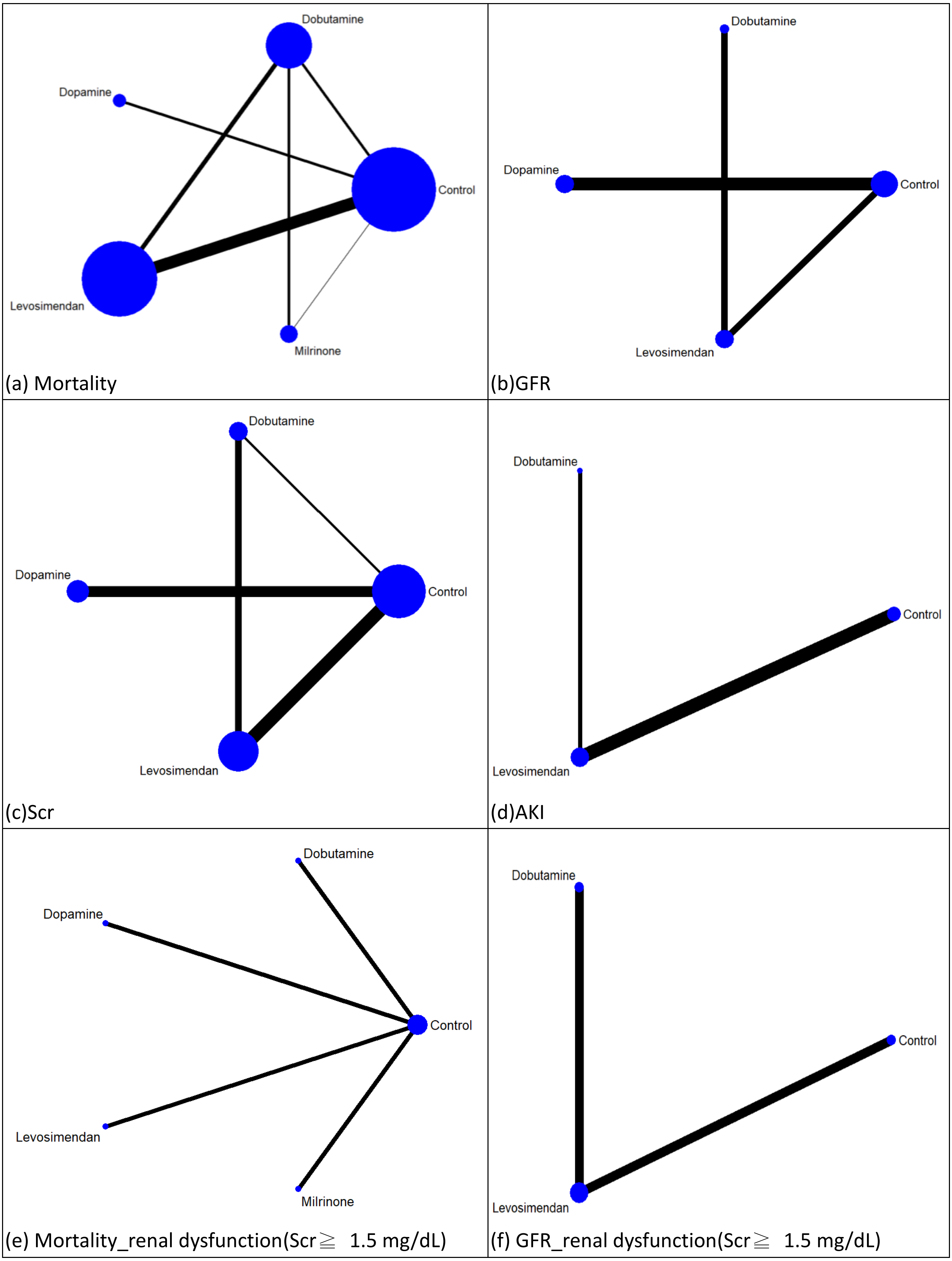


**Figure S1: Network plot for included therapies. The solid line represents as the direct comparisons. The thickness of the line represents the number of included trials.**

| Study | Domain 1 | Domain 2 | Domain 3 | Domain 4 | Domain 5 | Overall |
|---|---|---|---|---|---|---|
| Slawsky(2000) | + | + | + | + | + | + |
| Nieminen(2000) | + | − | + | + | + | − |
| Follath (2002) | + | + | + | + | + | + |
| Moiseyev VS (2002) | + | + | + | + | + | + |
| Adamopoulos (2006) | + | − | + | + | + | − |
| Flevari P (2006) | + | + | + | + | + | + |
| Parissis JT (2006) | + | − | + | + | + | − |
| Trikas(2006) | + | + | + | + | − | + |
| Mebazaa A (2007) | + | + | + | + | + | + |
| Yilmaz MB (2007) | + | + | + | + | + | + |
| Zemljic G (2007) | + | − | + | + | + | − |
| Mehmet (2009) | + | − | + | + | + | − |
| Kleber(2009) | + | + | + | + | + | + |
| Iyisoy A (2010) | + | − | + | + | − | X |
| Pasqui(2011) | + | + | − | + | − | X |
| Bonios MJ (2012) | + | − | + | + | + | − |
| Llorens(2012) | + | + | + | + | + | + |
| Hou ZQ (2013) | + | + | + | + | + | + |
| Husebye (2013) | + | + | + | + | + | + |
| Francesco (2014) | + | + | + | + | + | + |
| Jia (2014) | + | + | + | + | + | + |
| Jia (2015) | + | + | + | + | + | + |
| Saima Mushtaq (2015) | + | + | + | + | + | + |
| Josep (2018) | + | + | + | + | + | + |
| Lukas Lannemyr (2018) | + | + | + | + | + | + |
| Wang (2019) | + | − | + | + | + | + |
| Nanas(2004) | + | − | − | + | − | X |
| Robinson (1994) | + | + | − | + | − | X |
| Varriale (1997) | + | − | + | + | − | X |
| Giamouzis (2010) | + | + | + | + | + | + |
| Chen (2013) | + | + | + | + | + | + |
| Triposkiadis (2014) | + | + | + | + | + | + |
| Kamiya (2015) | + | − | + | + | − | X |
| Biddle (1987) | + | − | + | + | − | X |
| Karlsberg (1996) | + | − | + | + | − | X |
| Aranda (2002) | + | − | + | + | − | X |
| Cuffe (2002) | + | + | + | + | + | + |

**Figure S2. (a) Risk of bias summary: The judgements about each risk of bias item for each included study. Judgement as following: ⊗ means high risk; ⊖ means some concerns; ⊕ means low risk**

Domains:

Domain 1: Bias due to randomisation.

Domain 2: Bias due to deviations from intended intervention.

Domain 3: Bias due to missing data.

Domain4: Bias due to outcome measurement.

Domain 5: Bias due to selection of reported result

Figure S2.(a)

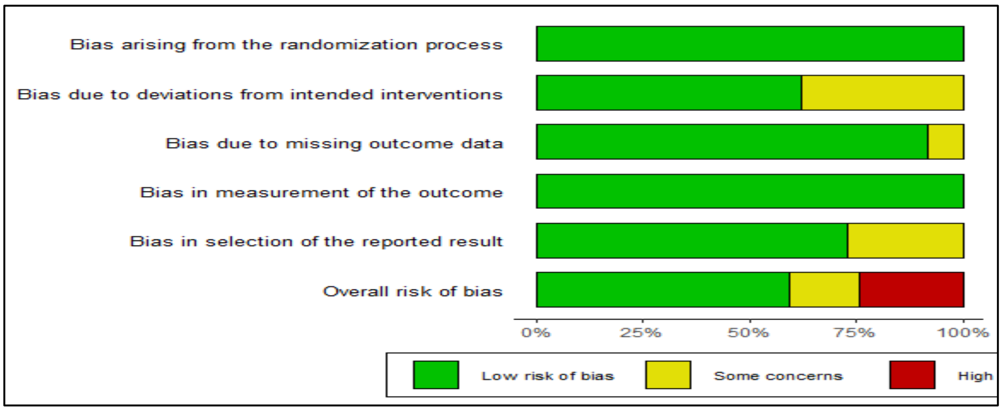


Figure S2.(b)

**Figure S2. (b) Risk of bias graph: The judgements about each risk of bias item reflected s percentages across all included studies**

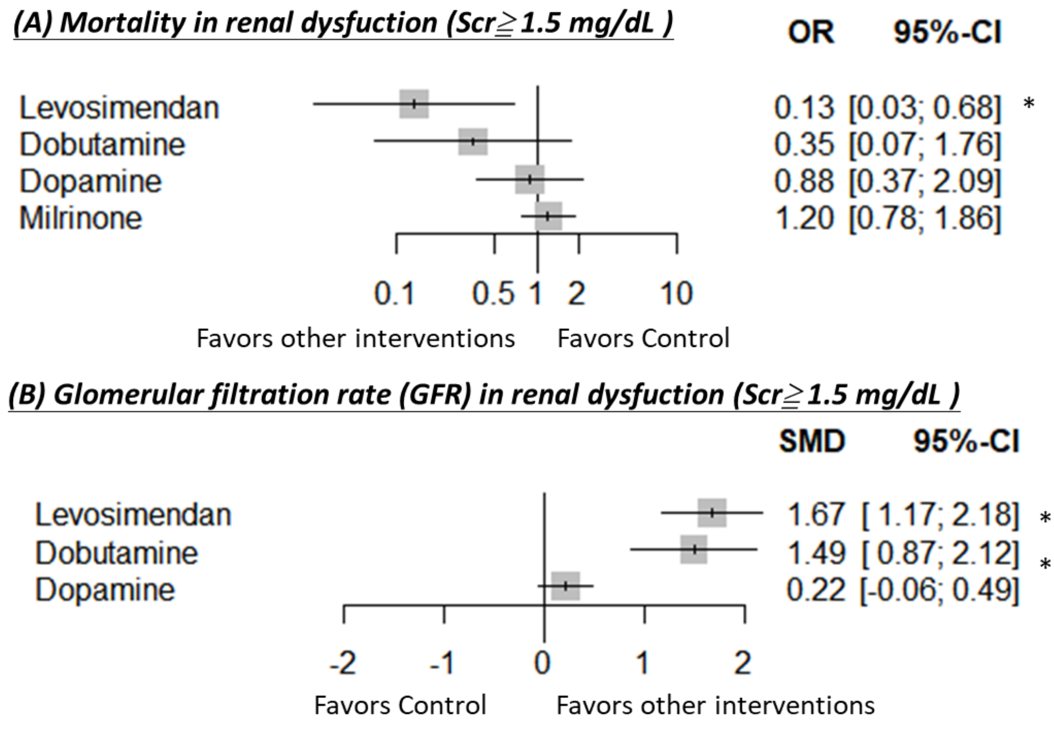


**Figure S3. Forest plots of subgroup in patients with renal dysfunction defined as Scr≥ 1.5 mg/dL. Interventions were compared with control for (A) mortality and (B) GFR. CI=credible interval. OR=odds ratio. SMD=standardized mean difference. *Significant results.**

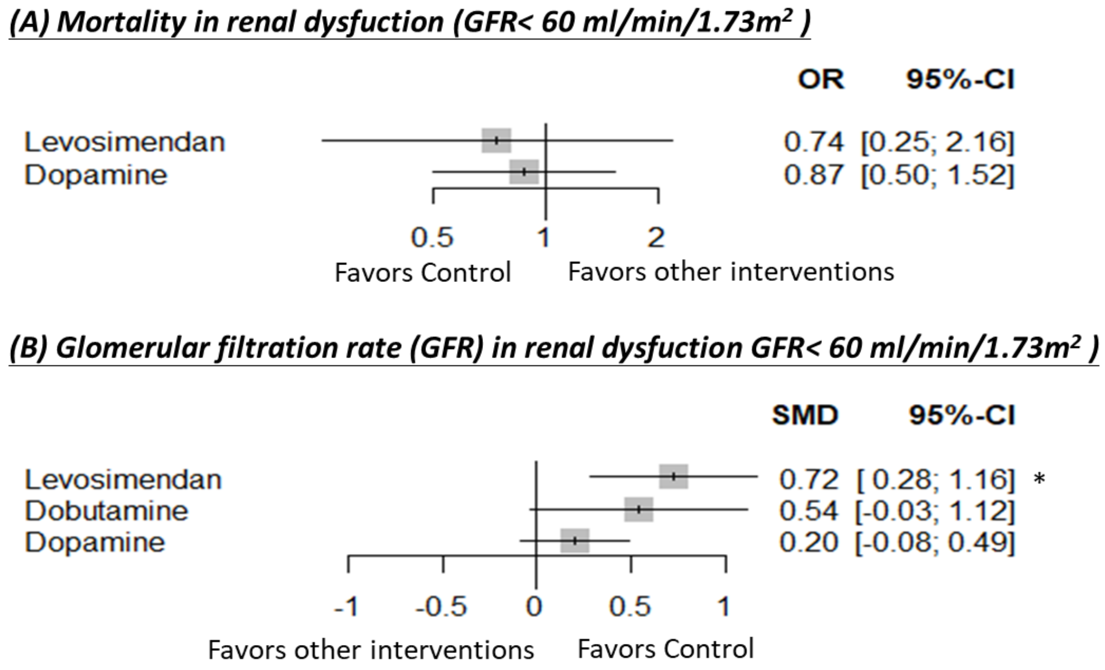


**Figure S4. Forest plots of subgroup in patients with renal dysfunction defined as GFR< 60 ml/min/1.73m$^2$. Interventions were compared with control for (A) mortality and (B) GFR. CI=credible interval. OR=odds ratio. SMD=standardized mean difference. *Significant results.**

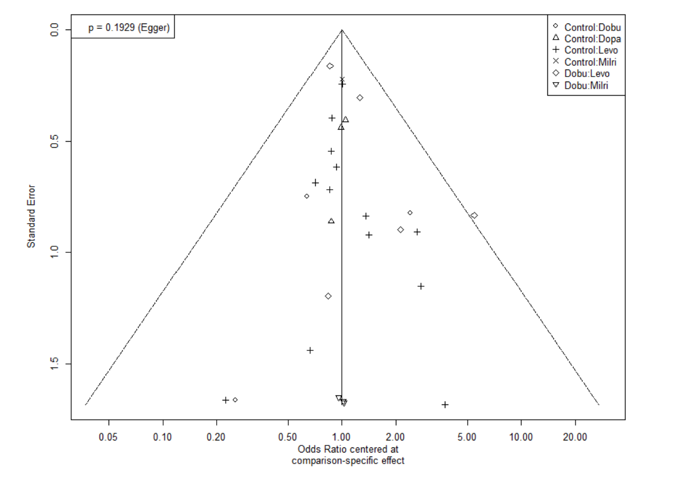


(a) Mortality

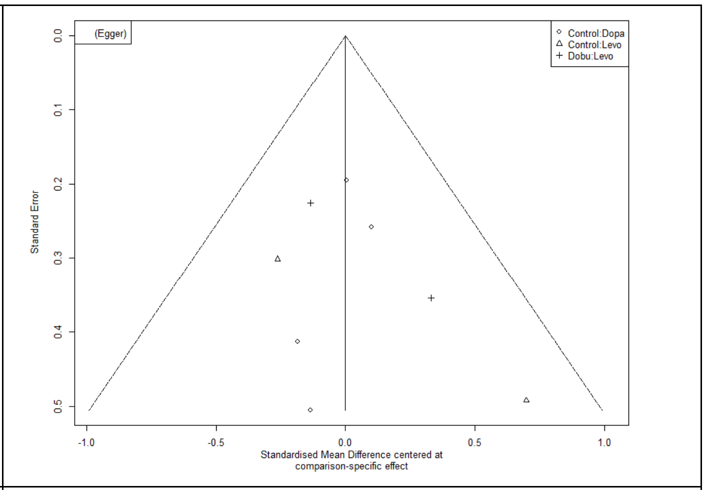


(b) GFR

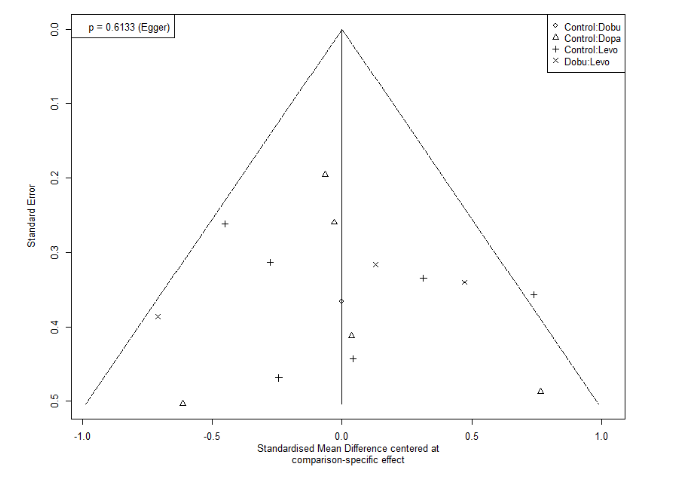


(c) Scr

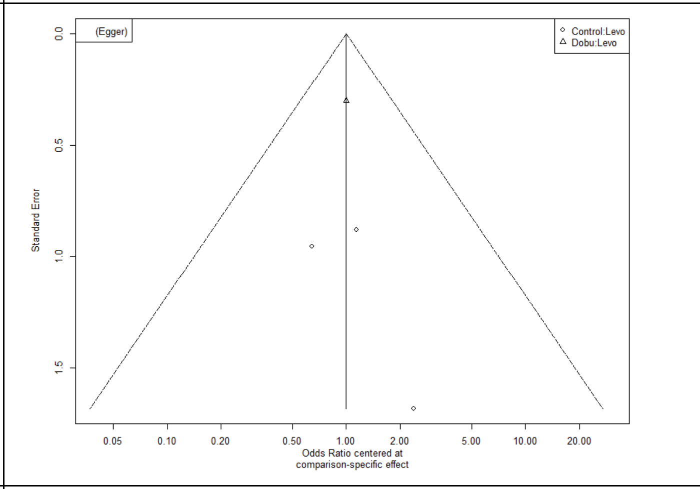


(d) AKI

**Figure S5. Funnel plot for each outcome. Each dot reflected a study; the vertical axis reflected the standard error and x-axis reflected the effect size of each study. Large studies distribute in the top of the plot, and smaller studies scatter toward the bottom of the plot. Dobu= Dobutamine; Dopa= Dopamine; Levo= Levosimendan; Milri= Milrinone**
